# Opportunities and challenges in combining immunotherapy and radiotherapy in esophageal cancer

**DOI:** 10.1007/s00432-023-05499-z

**Published:** 2023-11-20

**Authors:** Xinyu Zhang, Xinsheng Cai, Chaoguang Yan

**Affiliations:** 1 Weifang Hospital of Traditional Chinese Medicine, 666 Weizhou Road, Weifang, 261000 Shandong China; 2 Shandong University of Traditional Chinese Medicine, Jinan, 250000 Shandong China

**Keywords:** Immunotherapy, Radiotherapy, Radiotherapy combined with immunotherapy, Esophageal cancer, Immune checkpoint inhibitors, Low-dose radiotherapy

## Abstract

**Background:**

Immunotherapy has shown promise in the treatment of esophageal cancer, but using it alone only benefits a small number of patients. Most patients either do not have a significant response or develop secondary drug resistance. The combination of radiotherapy and immunotherapy appears to be a promising approach to treating esophageal cancer.

**Purpose:**

We reviewed milestone clinical trials of radiotherapy combined with immunotherapy for esophageal cancer. We then discussed potential biomarkers for radiotherapy combined with immunotherapy, including programmed cell death-ligand 1 (PD-L1) status, tumor mutation burden (TMB), tumor-infiltrating lymphocytes, ct-DNA, imaging biomarkers, and clinical factors. Furthermore, we emphasize the key mechanisms of radiation therapy-induced immune stimulation and immune suppression in order to propose strategies for overcoming immune resistance in radiation therapy (RT). Lastly, we discussed the emerging role of low-dose radiotherapy (LDRT) , which has become a promising approach to overcome the limitations of high-dose radiotherapy.

**Conclusion:**

Radiotherapy can be considered a triggering factor for systemic anti-tumor immune response and, with the assistance of immunotherapy, can serve as a systemic treatment option and potentially become the standard treatment for cancer patients.

## Background

Despite significant progress in the treatment of esophageal cancer, its efficacy remains unsatisfactory. Immunotherapy, particularly immune checkpoint inhibitors (ICIs), has emerged as a promising new treatment strategy, completely changing the treatment prospects for esophageal cancer. However, the use of single-agent ICI method is limited to a response rate of only around 20% (Kato et al. 2019; Kojima et al. 2020; Huang et al. 2020; Shen et al. 2022). To overcome this limitation, researchers have explored the combination of immunotherapy with other treatment modalities. Radiation therapy is a widely used method for esophageal cancer at various stages, and when combined with immunotherapy, it has garnered considerable attention. The KEYNOTE-001 study revealed significant efficacy of ICIs in patients who had previously received radiation therapy (Shaverdian et al. 2017). Through the synergistic effect of immunotherapy and radiation therapy, the aim is to enhance the immune response against cancer cells while precisely targeting the tumors. Although further extensive multicenter studies are needed to validate these findings, the combination of immunotherapy and radiation therapy represents a promising approach that can reshape the treatment paradigm for esophageal cancer, providing new hope for patients and further advancing the field of oncology.

Research has shown that immunotherapy enhances immune responses by enhancing co-stimulatory signals, restoring immune surveillance, and promoting the destruction of cancer cells. Co-stimulatory signals primarily involve positive co-stimulatory factors (CD27, CD28, CD137) and negative co-stimulatory factors (CTLA-4, PD-1, PD-L1) pathways (Rowshanravan et al. 2018; Hosseini et al. 2020). Tumor cells activate immune checkpoints by binding to PD-1 and PD-L1, which hinders antigen presentation and evades attacks from T cells (Zheng et al. 2021; Ai et al. 2020). Immune checkpoint inhibitors (ICIs) block the interaction between PD-1 and PD-L1, promote T cell activation, and enhance their anti-tumor efficacy. Previous studies have demonstrated that stereotactic body radiation therapy (SBRT), as a form of precise radiation therapy, can promote the release of tumor antigens, leading to a phenomenon known as the “abscopal effect” (Theelen et al. 2019). The combination of ICIs and radiotherapy enhances local therapeutic efficacy by promoting antigen release, improving antigen presentation, and modifying the tumor microenvironment (Sardaro et al. 2021).

The impact of radiotherapy on the immune response in esophageal cancer has been studied. Du et al. (2022) performed fractionated irradiation on two human esophageal cancer cell lines (KYSE450, OE21), with a dose of 30 Gy/10 F, administered 5 times per week, to study changes in immune activity. The study found that radiotherapy can regulate the expression of interferons and affect signaling pathways such as STING–IFNAR1–STAT1–IRF1, resulting in enhanced immune responsiveness. When the irradiation dose was 30 Gy/10 F, the immune response of the cells and the formation of micronuclei significantly increased. In addition, genes associated with the STING–IFNAR1–STAT1–IRF1 pathway, MHC class I and II, as well as PD-L1 expression, were significantly upregulated. Zhang et al. (2017) used a 6-MV linear accelerator to irradiate three esophageal squamous cell lines (KYSE30, TE1, CaEs17) at doses of 0, 2, 4, and 8 Gy. The study showed that the expression of PD-L1 on the surface of tumor cells increased in a dose-dependent manner after radiation exposure, possibly related to the secretion of gamma interferon by CD8 + T lymphocytes and activation of the JAK-STAT pathway (Dovedi et al. 2014). These findings emphasize the impact of radiotherapy on immune-related mechanisms and provide insights into exploring the combined use of radiotherapy and immunotherapy in the treatment of esophageal cancer.

The aforementioned preclinical studies indicate that radiotherapy plays a crucial role in upregulating the expression of PD-L1 in the tumor microenvironment, thereby enhancing the efficacy of immune checkpoint inhibitors. A study conducted by Lim et al. (2016) supports this notion, as it observed a significant increase in PD-L1 expression (*p* = 0.007) in locally advanced esophageal squamous cell carcinoma (ESCC) patients after neoadjuvant chemoradiotherapy, highlighting the potential of this combination therapy. However, due to limitations such as small sample size, strict patient selection, and short follow-up time, these findings still require further well-designed multicenter studies to validate them and establish optimal treatment strategies to improve clinical outcomes.

Esophageal cancer is a highly heterogeneous disease (Dinh et al. 2021; Yan et al. 2019; Li et al. 2023; Krug and Michl 2017), with patients exhibiting diverse biological, clinical, genetic, and environmental characteristics. Existing treatment approaches for esophageal cancer are based on average results from previous clinical trials and typically follow traditional strategies involving radiotherapy and immunotherapy. However, given the high heterogeneity of the disease, it is crucial to consider the specificity of individual patients and replace a "one-size-fits-all" approach with personalized methods when it comes to combination therapies.

This article provides a review of the current research status of combined radiotherapy and immunotherapy in the treatment of esophageal cancer, and explores various aspects related to its therapeutic efficacy. We investigate the mechanisms of effectiveness in this combination therapy and discuss the limitations of existing treatment strategies. Clearly, optimizing precision treatment is crucial for addressing the heterogeneity of esophageal cancer. By customizing treatment plans to accommodate the specific characteristics of each patient, better outcomes can be achieved. Further research in this field holds great promise for advancing the field and improving treatment outcomes for patients.

## Current status

In 2012, Postow et al. (2012) first reported a case of malignant melanoma in which a combination of ipilimumab and radiotherapy was used for treatment. Not only did the primary lesion completely regress, but even tumors outside the radiation field (such as right hepatic lymph nodes and spleen) also completely disappeared. In recent years, with the deepening of research on T cell regulation mechanisms, an increasing number of immune checkpoint inhibitors have been developed, showing remarkable efficacy in clinical studies targeting various malignancies (including tumors previously considered immune-resistant). These pioneering studies on combined radiotherapy and immunotherapy have spurred further clinical research in the field of esophageal cancer. Most recently, a phase II clinical trial, EC-CRT-001, conducted a single-arm study on locally advanced esophageal squamous cell carcinoma patients who were not eligible for surgical resection. The trial investigated the combination therapy of pembrolizumab (a PD-1 inhibitor) with chemoradiotherapy. Among the patients enrolled in the trial, 62% achieved complete remission, with a 1-year overall survival rate of 78.4% and a 1-year progression-free survival rate of 54.5%. Compared to traditional treatment methods, this approach has demonstrated significant efficacy and acceptable side effects for patients with locally advanced esophageal squamous cell carcinoma (Zhu et al. 2023). Another clinical trial, PALACE-1, treated 20 patients with resectable esophageal squamous cell carcinoma using preoperative pembrolizumab (PD-1 inhibitor) with concurrent chemoradiotherapy (PPCT) method. The results showed that 65% of patients experienced grade III or higher adverse events, with lymphocytopenia being the most common (92%). Among the 18 patients who underwent surgical treatment, the pathologic complete response (pCR) rate reached 55.6%. The study demonstrated that PPCT treatment has potential advantages in terms of safety and achieving pCR in resectable cases (Li et al. 2021). The results suggest that both trastuzumab and pembrolizumab treatments are safe and feasible adjuvant therapies for locally advanced esophageal cancer patients.

Systematic searches were performed in ClinicalTrial.gov databases up to June 2023 to gather information on clinical trials that explore the efficacy of the combination of radiotherapy and ICIs for the treatment of esophagus cancer. Table 1 presents a summary of the recent clinical trials of radiotherapy combined with immunotherapy. Currently, several prospective, multicenter, randomized clinical trials on radiotherapy combined with immune checkpoint inhibitors for the treatment of esophageal cancer are still ongoing. It is expected that in the near future, the safety and efficacy of combined radiochemotherapy with immune checkpoint inhibitors and neoadjuvant therapy will become clearer. This will contribute to further refinement and standardization of clinical radiochemotherapy combined with immunotherapy regimens.

**Table 1:** Clinical trials investigating the combination of radiotherapy and immune checkpoint inhibitors in esophageal cancer

ClinicalTrials.gov identifier	Trial phase	Participants	Radiotherapy regimen	Immunotherapy regimen	Chemotherapy regimen	Estimated/actual study completion date	Status
Neoadjuvant therapy							
NCT02735239	I/II	73	Standard care dose	Durvalumab 750 mg Q2W	Paclitaxel + carboplatin	June 16, 2022	Completed
NCT03044613	Ib	32	Standard care dose	Induction nivolumab 240 mg ± relatlimab 80 mg Q2W *2 Concurrent nivolumab 240 mg ± relatlimab 80 mg Q2W *3	Paclitaxel + carboplatin	February 2025	Active, not recruiting
NCT03165994	II	34	50.4GY/28F	Sotigalimab 0.3 mg/kg Q3W *3	Paclitaxel + carboplatin	April 30, 2024	Active, not recruiting
NCT03544736	I/II	30	Palliative: 20-50GY(2-4GY/F) Definitive: 50.4GY/28F Neoadjuvant: 41.4GY/23F	Palliative: nivolumab 240 mg Q2W/360 mg Q3W/480 mg Q4W (maximum 2 years); definitive: nivolumab 240 mg Q2W during radiotherapy, 480 mg Q4W (maximum 1 year); neoadjuvant: nivolumab 240 mg Q2W; postoperative: nivolumab 480 mg Q4W (maximum 1 year)	Paclitaxel + carboplatin	December 31, 2040	Active, not recruiting
NCT03792347	Ib	20	41.4GY/23F	Pembrolizumab 2 mg/kg Q3W *2	Paclitaxel + carboplatin	June 17, 2020	Completed
NCT03940001	I	20	41.4GY/23F	Sintilimab 200 mg Q3W	Paclitaxel + carboplatin	January 2031	Recruiting
NCT04229459	II	31	50.4GY/28F	Nivolumab 3 mg/kg Q2W *3	Cisplatin + 5-FU; cetuximab	June 2027	Recruiting
NCT04435197	II	143	41.4GY/23F	Pembrolizumab 200 mg Q3W *2	Albumin paclitaxel + carboplatin	December 2025	Recruiting
NCT04437212	II	20	41.4GY/23F	Neoadjuvant period: toripalimab 240 mg Q3W *2 Postoperative period: toripalimab 240 mg Q3W *4	Paclitaxel + cisplatin	December 31, 2023	Recruiting
NCT04568200	II	60	41.4GY/23F	Durvalumab 1500 mg Q3W *4	Paclitaxel + carboplatin	December 2023	Recruiting
NCT04644250	II	32	41.4GY/23F	Toripalimab 240 mg Q3W *3	Paclitaxel liposome + carboplatin	March 1, 2024	Recruiting
NCT04776590	II	30	41.4GY/23F	Tislelizumab 200 mg Q3W *3	Albumin paclitaxel + carboplatin	December 15, 2024	Recruiting
NCT04888403	II	45	41.4GY/23F	Induction toripalimab 240 mg *1, concurrent toripalimab 240 mg Q3W *4	Albumin paclitaxel + nedaplatin		
NCT04929392	II	24	41.4GY/23F	Pembrolizumab Q3W *2	Paclitaxel + carboplatin; lenvatinib	April 25, 2027	Recruiting
NCT04973306	II/III	176	41.4GY/23F	Tislelizumab 200 mg Q3W *2	Paclitaxel + carboplatin	July 2027	Recruiting
NCT04974047	II	70	40GY/20F	Tislelizumab 200 mg Q3W *3	paclitaxel + cisplatin; cisplatin + 5-FU	May 30, 2026	Active, not recruiting
NCT05043688	II	204	41.4GY/23F	Neoadjuvant period: camrelizumab 200 mg Q3W *2 Postoperative period: camrelizumab 200 mg Q3W (maximum 1 year)	Albumin paclitaxel/paclitaxel + carboplatin	March 6, 2026	Not yet recruiting
NCT05176002	I/II	26	41.4GY/23F	Camrelizumab	NA	September 2024	Recruiting
NCT05189730	II	80	40GY/20F	Neoadjuvant period: tislelizumab 200 mg Q3W *2; maintenance period: tislelizumab 200 mg Q3W (maximum 1 year)	Paclitaxel + carboplatin	December 31, 2023	Recruiting
NCT05355168	I/II	57	41.4 GY/23F	Camrelizumab	Paclitaxel + carboplatin; nimotuzumab	December 1, 2025	Recruiting
NCT05424432	II	63	30GY/12F	Toripalimab 240 mg Q3W *2	Paclitaxel + carboplatin	December 31, 2024	Recruiting
NCT05507411	II	100	41.4GY/23F	Camrelizumab 200 mg Q3W	Albumin paclitaxel + carboplatin	April 2027	Recruiting
NCT05541445	Ib/II	40	44GY/22F	Neoadjuvant period: induction pembrolizumab + chemotherapy 200 mg Q3W *2, Sequential pembrolizumab + chemoradiotherapy 200 mg Q3W *2; postoperative period: pembrolizumab 200 mg Q3W (maximum 1 year)	Albumin paclitaxel + cisplatin	December 1, 2024	Recruiting
NCT05650216	II	50	Primary lesion and adjacent lymph nodes: 41.4GY/23F; Abscopal lymph node 2GY/4F	Neoadjuvant period: camrelizumab 200 mg Q3W *2; postoperative period: camrelizumab 200 mg Q3W (maximum 1 year)	Albumin paclitaxel + carboplatin	December 25, 2024	Not yet recruiting
Adjuvant therapy							
NCT03322267	II	26	adjuvant 18-26GY/10-13F	Pembrolizumab 200 mg Q3W *18	Cisplatin	June 30, 2025	Recruiting
NCT04741490	NA	20	adjuvant 45-55GY (1.8–2.0GY/F)	Camrelizumab 200 mg Q3W *6	NA	August 2023	Recruiting
NCT05103501	II	54	NA	Pembrolizumab 200 mg Q3W (maximum 2 years)	Cisplatin + 5-FU	September 30, 2024	Not yet recruiting
Definitive therapy							
NCT03222440	Ib	20	54-60GY/30F	Camrelizumab 200 mg Q2W *16	NA	November 1, 2019	Completed
NCT05515315	II	93	50-60GY/25-30F	Tislelizumab 200 mg Q3W	Albumin paclitaxel + nedaplatin	August 8, 2025	Not yet recruiting
NCT03278626	I/II	44	50.4GY/28F	Nivolumab 240 mg Q2W	Paclitaxel + carboplatin	March 28, 2022	Terminated
NCT03437200	II	130	50GY/25F	Nivolumab 240 mg Q2W (maximum 1 year); Ipilimumab 1 mg/kg Q6W (maximum 1 year)	Oxaliplatin + leucovorin + 5-FU	October 7	Terminated
NCT03777813	II	120	Macroscopic disease: 50GY/25F; adjacent peri tumoral mucosis and prophylactic lymph node: 45GY/25F	Durvalumab 1500 mg Q4W (maximum 1 year)	Oxaliplatin + leucovorin + 5-FU	September 1, 2024	Recruiting
NCT03957590	III	370	50.4GY/28F	Tislelizumab 200 mg Q3W (maximum 2 years)	Paclitaxel + cisplatin	December 30, 2024	Active, not recruiting
NCT04005170	II	42	50.4GY/28F	Toripalimab 240 mg Q3W (maximum 1 year)	Paclitaxel + cisplatin	July 31, 2022	Completed
NCT04210115	III	700	50/25F 60GY/30F	Pembrolizumab 200 mg Q3W *8 followed by 400 mg Q6W *5	Cisplatin + 5-FU; oxaliplatin + leucovorin + 5-FU	April 15, 2027	Recruiting
NCT04426955	III	396	50.4GY/28F	Camrelizumab 200 mg Q3W	paclitaxel + cisplatin	December 2022	Active, not recruiting
NCT04550260	III	600	50-64GY	Durvalumab (maximum 2 years)	Cisplatin + 5-FU; cisplatin + capecitabine	November 30, 2026	Recruiting
NCT04821778	III	2000	50-66GY/25-30F	Anti-PD-1/PD-L1 Antibody	Paclitaxel/platinum/5-FU	December 31, 2025	Recruiting
NCT04844385	II	83	60GY/24F	Toripalimab 240 mg Q3W *2	Albumin paclitaxel + nedaplatin; capecitabine	July 2024	Recruiting
NCT04851132	II	33	59.92GY/28F	Durvalumab 1000 mg Q3W *18	NA	June 2023	Recruiting
NCT05520619	II	114	50.4GY/28F	Tislelizumab 200 mg Q3W *4 ± maintenance tislelizumab 200 mg Q3W *12	Paclitaxel + cisplatin	July 31, 2026	Recruiting
NCT05621707	II	50	NR	Sintilimab 200 mg Q3W (maximum 1 year)	Albumin paclitaxel + carboplatin	November 19, 2026	Not yet recruiting
NCT05624099	II	226	50GY/30F	Camrelizumab 200 mg Q3W	Paclitaxel + platinum	December 31, 2027	Not yet recruiting
Consolidative therapy							
NCT04212598	II	40	50.4GY/28F, patients with residual disease boost to 61.2 Gy/34F	Sintilimab 200 mg Q3W (maximum 1 year)	NA	December 3, 2023	Recruiting
NCT04390945	II	62	50–50.4GY/25-28F	Camrelizumab 200 mg Q2W	Capecitabine	August 2024	Recruiting
NCT04514835	II	44	50–50.4GY/25-28F	Sintilimab 200 mg Q3W (maximum 1 year)	Cisplatin + capecitabine	January 31, 2023	Not yet recruiting
NCT04543617	III	750	standard care dose	Atezolizumab 1200 mg Q3W *17; tiragolumab 600 mg Q3W *17	Platinum-based	June 27, 2025	Recruiting
NCT04821765	II	35	50-60GY (1.8-2GY/F or 3-4GY/F)	Tislelizumab 200 mg Q3W (maximum 1 year)	Albumin paclitaxel + cisplatin	September 30, 2023	Recruiting
NCT05183958	II	118	SBRT, 8GY/F 3-5F; Conventional ≥ 40GY	Camrelizumab 200 mg Q3W (maximum 2 years)	Paclitaxel + platinum; cisplatin + 5-FU; capecitabine + cisplatin	December 1, 2025	Not yet recruiting
NCT05512520	II	126	45–50.4GY/25-28F	Anti-PD1 Q3W	Fluoropyrimidine or taxane-based platinum doublet; capecitabine	September 30, 2025	Recruiting
NCT05547828	II	20	40GY/20F (primary tumor and metastases)	Tislelizumab 200 mg Q3W	Albumin paclitaxel	December 20, 2024	Not yet recruiting
NCT05628610	II	130	50GY/30F	Tislelizumab 200 mg Q3W	Paclitaxel + platinum	December 1, 2027	Not yet recruiting

## Patient choice and biomarkers: updates and challenges

In recent years, the combined application of radiation therapy and immunotherapy has shown promising prospects in the treatment of tumors. However, it is important to recognize that this combination therapy may not yield the same clinical outcomes for all patients. Several factors contribute to the heterogeneity of immune therapy responses, including clinical and pathological factors, age, physical condition, and disease burden. To address this issue, researchers have been exploring the use of biomarkers to predict the efficacy of immune radiotherapy and to classify patients into different response and risk levels, ultimately aiding in the selection of personalized treatment strategies. We have summarized emerging biomarkers for predicting the efficacy of immune radiotherapy.

### PD-L1

PD-L1 is currently considered the most widely accepted biomarker for predicting the efficacy of immunotherapy (Luchini et al. 2019; Gibney et al. 2016). For patients with high expression of PD-L1, standalone immunotherapy appears to provide a higher treatment effect, suggesting that they may not need radiation therapy to control the disease. Conversely, patients with low expression of PD-L1 may benefit from radiation therapy to achieve disease control, as their response to standalone immunotherapy may be lower (Theelen et al. 2021). The combination of radiation therapy and immunotherapy for patients with low PD-L1 expression may improve the treatment effect by enhancing disease control and bolstering immune response. Considering the individualized treatment strategy based on PD-L1 expression levels and the potential benefits of radiation therapy helps optimize treatment decisions and improve patient outcomes.

### TMB

Tumor mutation burden (TMB) has the potential to serve as an important biomarker for predicting the response to immunotherapy, and its correlation with response rates to anti-PD-1 or anti-PD-L1 treatment has been confirmed in several types of tumors (Yarchoan et al. 2017; Lu et al. 2019; Chan et al. 2019). In numerous comparative trials evaluating the efficacy of immunotherapy and chemotherapy, TMB has shown prospects as an independent predictive biomarker (Yan et al. 2022; de Klerk et al. 2021). However, the role of TMB in predicting the efficacy of radiation therapy remains uncertain. In clinical studies exploring the combination of immunotherapy and radiation therapy for esophageal cancer, the assessment of TMB has not yet been uniformly incorporated. Further research is needed to determine the impact of TMB on radiation therapy outcomes and the potential inclusion of TMB in the decision-making for immune-radiation combination treatments. Understanding the relationship between TMB, immunotherapy, and radiation therapy can strengthen the selection strategies and optimize treatment methods for patients with esophageal cancer.

### Tumor-infiltrating lymphocytes

Immune checkpoint inhibitors require lymphocytes to be adequately infiltrated in tumor tissue to exert their anti-tumor effects. Radiation therapy can stimulate local innate and adaptive immune responses by increasing the infiltration of tumor-infiltrating lymphocytes (TILs). Therefore, the composition, density, functional status, and tissue characteristics of TILs can serve as predictive indicators of the response to radiation and immunotherapy (Miller et al. 2019). CD8 + T cells play a crucial role in tumor clearance, and their infiltration is positively correlated with improved prognosis. Studies have shown that higher levels of PD-1 + CD8 + T cells in the tumor microenvironment can predict better outcomes for esophageal cancer patients receiving PD-1/PD-L1 inhibitor therapy (Zhang et al. 2021; Wu et al. 2020). Conversely, myeloid-derived suppressor cells (MDSCs), regulatory T cells (Tregs), tumor-associated macrophages, and neutrophils are enriched in the tumor microenvironment, negatively impacting prognosis due to their inhibitory effects on immune response (Kalathil and Thanavala 2021; Shang et al. 2015). Direct assessment of TILs remains challenging due to limited availability of histological material obtained from biopsies and the variability of the microenvironment at different metastatic sites. Ongoing research focuses on establishing and validating guidelines for TIL assessment.

### ctDNA

Circulating tumor DNA (ctDNA) originates from tumor cells and contains genomic information of the tumor (Thurin et al. 2020). ctDNA can be a reliable tool for prognostic stratification after radiotherapy/radiochemotherapy. ctDNA assessment has potential in selecting appropriate candidates for adjuvant therapy after surgery and determining consolidation therapy after radiotherapy/radiochemotherapy (Wang et al. 2022a). Recent studies have shown that ctDNA levels can predict disease progression after immunotherapy and can be used to monitor early response to anti-PD-1 immunotherapy, closely correlating with progression-free survival (PFS) and overall survival (OS) (Cabel et al. 2017; Khagi et al. 2017; Keller et al. 2019). These findings suggest that ctDNA has the potential to serve as a non-invasive monitoring tool for evaluating treatment response and prognosis in patients with esophageal cancer undergoing radiotherapy combined with immunotherapy.

The predictive value of ctDNA in radiotherapy is still unclear. Existing evidence suggests that ctDNA levels increase shortly after radiation (Kageyama et al. 2018) but data from related studies are limited, making it impossible to reach a consensus on its predictive value. Further research and more evidence are needed to determine the true potential of ctDNA as a predictive biomarker for radiotherapy.

### Imaging biomarkers

Functional imaging techniques such as PET-CT and MRI play a crucial role in predicting treatment response and evaluating prognosis in cancer patients. Optimizing these techniques and parameters is essential for improving prediction accuracy (Sakin et al. 2022; Lee et al. 2021; Borggreve et al. 2018). Clinical trials have demonstrated the efficacy of imaging responses in guiding treatment decisions (Goodman et al. 2021). Based on standard uptake values (SUV), tumor metabolic volume (MTV), and tumor glycolysis (TLG) determined by 18F-fluoro-2-deoxyglucose (18F-FDG) positron emission tomography (PET), they are associated with high levels of PD-L1 and the expression of PD-1, CD8, CD163 (tumor-associated macrophages), and Foxp3 (Tregs) TILs (tumor-infiltrating lymphocytes), suggesting the potential of metabolic parameters as prognostic biomarkers (Castello et al. 2020; Takada et al. 2017). Therefore, increasing research attempts to validate the predictive role of tumor metabolic variables in immunotherapy (Castello et al. 2020; Seban et al. 2020). By combining functional imaging with other biomarkers and ongoing research, personalized treatment for cancer can be advanced.

### Clinical factors

Advanced esophageal cancer exhibits heterogeneity in terms of organ metastasis and lymph node involvement, resulting in differences in the effectiveness of radiotherapy treatment. For patients with advanced esophageal cancer, in addition to standard treatment, active primary tumor radiotherapy has also shown positive results (Guttmann et al. 2017). Real-world studies have shown that the addition of radiotherapy to immunotherapy can significantly prolong the overall survival (OS) of patients with locally recurrent esophageal cancer. In the overall patient cohort, there was no statistically significant difference in progression-free survival (PFS) and OS between patients who received radiotherapy and those who did not (Wu et al. 2023), highlighting the importance of patient selection. Advanced esophageal cancer with limited metastasis and regional lymph node recurrence has a better prognosis, and the combination of active local and systemic treatments may provide an opportunity for curative treatment, which should be considered when choosing treatment options for patients. Decision tree models have been used to classify oligometastatic esophageal cancer into different risk groups, identifying high-risk patients who may benefit from early intensified treatment such as immunotherapy and concurrent chemoradiotherapy (Shi et al. 2022). Overall, the research results emphasize the importance of tailoring treatment plans based on individual characteristics of patients with advanced esophageal cancer, including the extent of organ metastasis, lymph node involvement, and recurrence patterns. Active primary tumor radiotherapy, combined with local and systemic treatments, offers a promising strategy for improving the prognosis of specific patients.

## The issues and challenges of radiotherapy combined with immunotherapy in the clinical treatment of esophageal cancer

Despite the combination of radiotherapy and immunotherapy being regarded as an effective new strategy for cancer treatment, its applicability remains somewhat controversial. Relevant research indicates that multiple factors may influence the efficacy of radiotherapy combined with immunotherapy.

### The toxicity and safety of combination therapy are yet to be clarified

A large number of clinical studies have found that the use of immunomodulatory agents alone can cause serious adverse reactions, including fulminant myocarditis with rhabdomyolysis, immune-related pneumonia, and interstitial pneumonia (Brown et al. 2021). In combination therapy, the incidence of high-grade adverse events ranges from 20 to 50%, with lymphocyte reduction being the most common, followed by esophagitis, anastomotic leak, and esophageal fistula (Duan et al. 2023; Wang et al. 2022b; Wu et al. 2022; Sha et al. 2020; Zhou et al. 2021; Voong et al. 2019). According to data from the US Food and Drug Administration, the incidence of grade 3–4 pneumonia in patients who did not receive radiotherapy, received immune checkpoint inhibitors (ICI) within 90 days after radiotherapy, and received ICI more than 90 days after radiotherapy was 1.1%, 1.9%, and 1.2%, respectively. Although combining radiotherapy with ICI may increase the incidence of severe radiation pneumonitis, the absolute increase percentage is small, indicating that ICI administration within 90 days after radiotherapy appears to be safe (Anscher et al. 2022). In patients receiving neoadjuvant chemoradiotherapy with or without immunotherapy, there was no statistical difference in major complications (such as pulmonary complications, anastomotic leak, and other complications) as well as the risk of death or readmission (Sihag et al. 2021). However, it has been reported that 61% of patients with a history of immune-related adverse events developed grade 2 or higher radiation pneumonitis following radiotherapy, and 83% of patients with previous ICI-associated pneumonia developed grade 2 or higher radiation pneumonitis (Shaverdian et al. 2020). A retrospective study also found that combining radiotherapy with anti-PD-1 increased the incidence of esophageal perforation (18% vs. 3.1%, *p* = 0.002) (Peng et al. 2021). Although most clinical trials consider the safety of combination therapy acceptable, the enrolled population usually has lower tumor burden and better performance status, which may not accurately reflect the occurrence of immune-related adverse events in routine clinical practice.

### The sequential regimen of combined radiotherapy and immunotherapy is still uncertain.

The integration of radiotherapy with immune checkpoint inhibitors (ICI) is a promising approach in cancer treatment, and various combination therapy modes have been explored. The three main approaches include synchronous ICI and radiotherapy, consolidation therapy with ICI after radiotherapy, and radiotherapy after induction with ICI. The theoretical separation of radiotherapy and ICI may potentially reduce adverse reactions. However, determining the optimal timing and interval between radiotherapy and ICI remains a challenge. Limited data are available for combined therapy in esophageal cancer. In this case, insights from lung cancer treatment can guide decision-making. The PACIFIC study demonstrated a slight improvement in outcomes for patients who started using Durvalumab within 2 weeks after radiotherapy (Antonia et al. 2018). Similarly, real-world PACIFICC-R study showed better prognosis for patients who initiated Durvalumab treatment within 42 days compared to those with delayed initiation (Girard et al. 2023). Meta-analysis suggests that the interval between radiotherapy and Durvalumab exceeds 42 days in real-world practice, with no significant impact on progression-free survival (PFS) or overall survival (OS) at 12 months (Wang et al. 2022c). Ongoing clinical trials for consolidation therapy in esophageal squamous cell carcinoma typically adopt a 6-week interval between the end of radiotherapy and the start of ICI. In cases involving esophagectomy and adjuvant immunotherapy, a longer interval may be required for immune system recovery (Kelly et al. 2021). It should be noted that the timing of initiating ICI after neoadjuvant chemoradiotherapy and curative surgery affects survival benefits. In patients with recurrent metastatic esophageal squamous cell carcinoma, median PFS and OS were prolonged in patients who received radiotherapy within 90 days before or after immunotherapy treatment (Wu et al. 2023). A multicenter retrospective study in Italy found that an interval of less than 7 days between stereotactic body radiotherapy (SBRT) and ICI prolonged OS, with slightly higher but manageable toxicity (Duan et al. 2023; Scoccianti et al. 2021). However, the most effective regimen for ICI combined with radiotherapy remains unclear and may vary depending on specific multimodal treatments and cancer types.

The sequencing of radiotherapy and immunotherapy is a key aspect of the treatment plan in radioimmunotherapy. In mouse models, simultaneous or short-term administration of PD-1 inhibitors after local radiotherapy has shown potential in enhancing intra-tumoral expansion of multifunctional CD8 + T cells and reducing peripheral CD8 + T cell death, thereby generating a more favorable systemic anti-tumor response and distant effects (Dovedi et al. 2014; Wei et al. 2021). Patients who receive immune checkpoint inhibitors (ICI) after stereotactic body radiotherapy (SBRT) or stereotactic radiosurgery (SRS), or concurrently with these treatments, also show improved outcomes (Woody et al. 2022; Kiess et al. 2015; Schoenfeld et al. 2015), possibly due to the release of new antigens induced by radiotherapy and increased PD-L1 expression. However, some studies have found no statistically significant difference between simultaneous and sequential administration (Welsh et al. 2019). Further research is needed to determine the optimal treatment sequence for different combinations and types of tumors.

Most studies tend to administer immune checkpoint inhibitors (ICI) simultaneously with or sequentially after radiotherapy. However, there are reports indicating that post-radiotherapy administration of anti-PD-1 agents in esophageal cancer may increase the risk of perforation and requires close monitoring (Peng et al. 2021). For patients with specific characteristics such as ulcerative, large, thin-walled, or invasion into major blood vessels, considering induction immunotherapy as first-line treatment may be an option. Induction immunotherapy can help alleviate symptoms, improve nutritional status, reduce irradiated volume, minimize side effects, and provide better protection to surrounding normal tissues.

In summary, although the integration of radiotherapy and immune checkpoint inhibitors (ICI) holds promise in cancer treatment, determining the optimal timing and interval between these modalities remains an ongoing area of research. Developing individualized treatment plans based on specific cancer types, neoadjuvant or adjuvant treatment settings, and patient characteristics is crucial for maximizing treatment efficacy.

### There is currently no standardized approach for determining the optimal dose and fractionation of radiotherapy in combination therapy

There has been ongoing controversy over the optimal dosage for esophageal cancer radiation therapy. However, in recent years, with the strengthening of systemic therapy, several large-scale phase III studies have compared the efficacy and safety of 50 Gy radiation dosage for esophageal squamous cell carcinoma with higher dosages (Hulshof et al. 2021; Xu et al. 2022; Minsky et al. 2002; Crehange et al. 2021). Currently, 50 Gy is the recommended dosage for radical radiotherapy for esophageal squamous cell carcinoma (Ajani et al. 2023). Guidelines suggest a radiation dosage range of 41.4–50.4 Gy for neoadjuvant radiotherapy. Studies have shown that higher radiation doses do not necessarily improve treatment outcomes or patient prognosis (Worrell et al. 2020). The CROSS (Chemoradiotherapy for Oesophageal Cancer followed by Surgery Study) regimen, with a dosage of 41.4 Gy administered in 23 fractions, has been widely adopted in neoadjuvant therapy due to its favorable effects. The SCALE study explored a shorter neoadjuvant radiotherapy course of 30 Gy/12F and achieved a 55% pathological complete response (pCR) rate (Jiang et al. 2022). In the NICE-RT study, the primary lesion and adjacent lymph nodes were conventionally fractionated with 41.4 Gy/23 fractions, while the distant lymph nodes received low-dose radiation of 0.5 Gy four times. For metastatic esophageal squamous cell carcinoma, radical or neoadjuvant radiotherapy regimens are commonly used for the primary lesion. Treatment strategies for metastatic lesions vary, including attempts at low-dose radiation (Herrera et al. 2022a), different dosage combinations, and SBRT/SRS techniques. The combination of high-dose and low-dose treatments (20–70 Gy, 3–12.5 Gy per session; 1–10 Gy, total dose of 0.5–2 Gy per session) has been shown to potentially reverse immune resistance after immunotherapy.

## Overcoming immunotherapy resistance with radiotherapy: updates and challenges

**Fig. 1:**
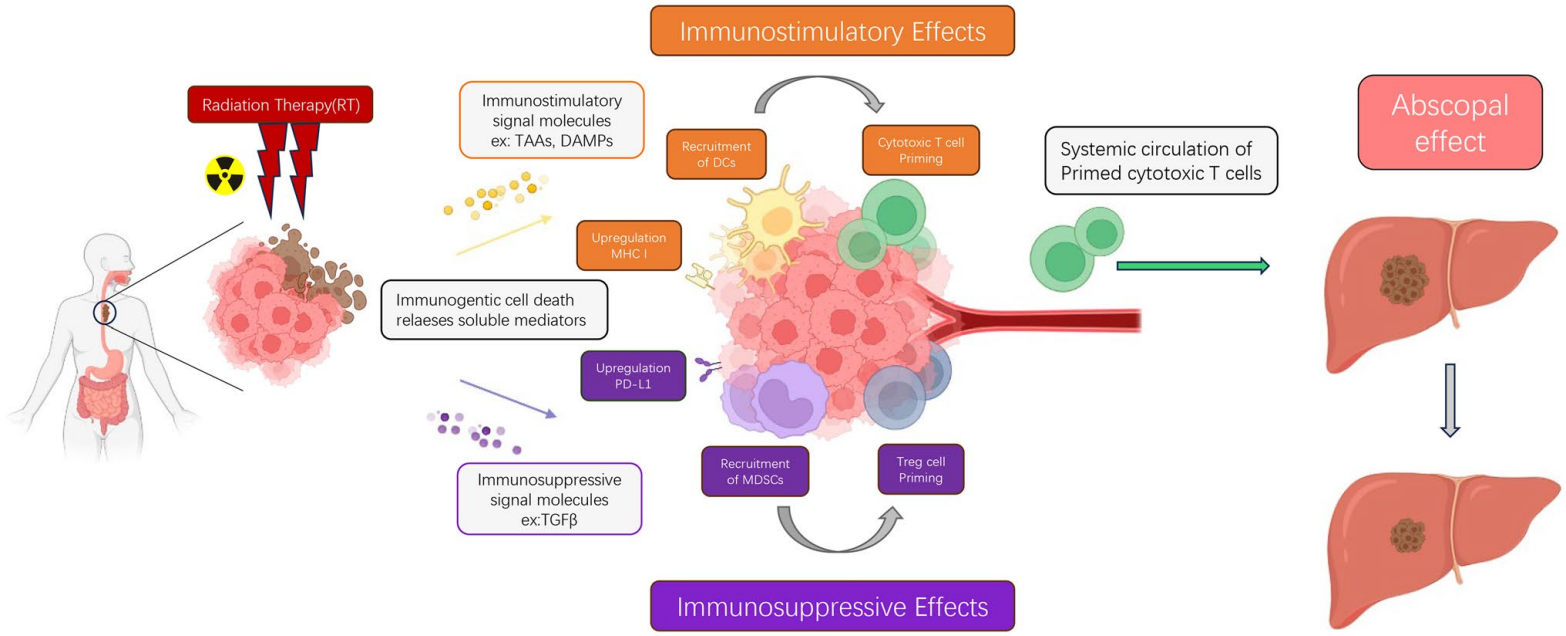
Effects of RT on the immune system. RT causes immunogenic cell death releases soluble mediators including cytokines, chemokines, tumor-associated antigens (TAAs), and damage-associated molecular patterns (DAMPs) inducing immunosuppressive and immunostimulatory effects. Additionally, radiation therapy can also generate abscopal effect, affecting immune responses beyond the irradiated site

Radiation therapy (RT) can directly destroy tumor cells and induce immune-mediated anti-tumor effects, as well as improve the tumor microenvironment (Formenti and Demaria 2009; Burnette et al. 2012; Rückert et al. 2021a; Brix et al. 2017; Meric-Bernstam et al. 2021). Localized radiation therapy can trigger tumor cell death, while releasing cytokines, chemokines, tumor antigens, and damage-associated molecular patterns (DAMPs) signals. These signals can enhance the function of antigen-presenting cells (APCs), activate tumor-specific T cell immunity, and promote APC recruitment. In addition, localized radiation therapy can also promote the expression of major histocompatibility complex (MHC) and loading of tumor antigens onto APCs. These antigen-loaded APCs will migrate to draining lymph nodes, stimulating the activation of immune responses, enhancing T cell proliferation, and improving their ability to recognize tumors (Formenti and Demaria 2009, 2013; Ahmed et al. 2013; Voorwerk et al. 2019). However, radiation therapy may also induce immune suppression in the tumor microenvironment by activating immune inhibitory factors such as transforming growth factor-beta (TGF-β), regulatory T cells (Tregs), tumor-associated macrophages (TAMs), and myeloid-derived suppressor cells (MDSCs). At the same time, the expression of programmed cell death-ligand 1 (PD-L1) in tumor cells is upregulated, which is closely related to the phenomenon of immune suppression in the tumor site (Yin et al. 2019; Zhai et al. 2022; Wang and Haffty 2018; Tagliaferri et al. 2022; Kho et al. 2021) (Fig. 1). Understanding the dual mechanism of localized radiation therapy in tumor immunity is crucial for developing strategies to optimize treatment efficacy and enhance overall anti-tumor immune responses.

In the treatment of oligometastatic lesions in advanced esophageal cancer with SBRT, a phenomenon known as the "abscopal effect" (Zhao et al. 2018) has been observed, where non-irradiated lesions also show shrinkage or regression. In recent years, a significant amount of research has been focused on studying the potential of combining radiation therapy with immunotherapy to induce the abscopal effect (Ngwa et al. 2018; Liu et al. 2018; Kodet et al. 2021; Wang et al. 2020; Buchwald et al. 2018). The abscopal effect is based on radiation therapy killing tumor cells, leading to the release of tumor-associated antigens (TAAs), which are subsequently captured by dendritic cells (DCs). DCs activate CD8 T cells, induce systemic immune responses, control tumor proliferation, and generate therapeutic effects in distant sites (Hu et al. 2017; Siva et al. 2015; Weichselbaum et al. 2017). Understanding and harnessing the abscopal effect may bring revolutionary changes to the treatment of advanced cancer, potentially improving outcomes and expanding disease control.

Higher single radiation doses may better support immune-induced mechanisms (Marconi et al. 2017), but they can also lead to serious adverse effects such as esophageal bleeding and perforation. This high-dose radiation in a single fraction is not suitable for the treatment of primary tumors in esophageal cancer. Therefore, currently, conventional low-dose radiation therapy is preferred in the treatment of esophageal cancer to minimize severe adverse effects.

The ongoing study NCT02642809 (NCT02642809: Pembrolizumab With Locally Delivered Radiation Therapy for the Treatment of Metastatic Esophageal Cancers) is a Phase I study conducted at a single institution, using an open-label design and a single dose level. Its primary objective is to evaluate the tolerability of high-dose radiotherapy combined with pembrolizumab in the treatment of metastatic esophageal cancer. The study utilizes hypofractionated radiotherapy (16 Gy in 2 fractions, with a 7–10 day interval between fractions) followed by pembrolizumab (200 mg, once every 3 weeks, starting within 1 week after completion of the short-range radiotherapy, and continuing for 8 months). The aim is to deliver an adequate dose of radiation to induce the release of tumor-derived antigens and trigger anti-tumor immune responses. The tolerability of the treatment will be measured by the incidence of treatment-related adverse events (TRAEs). This study aims to promote the use of immune-combined radiotherapy as a unique strategy for the treatment of advanced esophageal cancer. Conventional low-dose radiotherapy combined with immunotherapy for primary tumors and oligometastatic radiotherapy combined with immunotherapy for late-stage tumors may become the main treatment strategies for esophageal cancer.

Clinical pre-studies have shown that the phenomenon of distant tumor regression, known as the abscopal effect, occurs in immunocompetent environments rather than in immunocompromised conditions (Stone et al. 1979; Park et al. 2014). The research results emphasize the critical role of anti-tumor immunity as a key factor influencing the effectiveness of radiation therapy. In a mouse model of fibrosarcoma, immunocompetent hosts were able to control the tumor at lower radiation doses compared to immunodeficient hosts (Stone et al. 1979). Similarly, in another study using a mouse model of melanoma (B16 tumor), immunocompetent hosts responded to high-dose radiation, while immunodeficient hosts showed no response to radiotherapy and had a higher propensity for tumor metastasis. This evidence highlights the importance of anti-tumor immunity in mediating the abscopal effect (Lee et al. 2009).

Different immune therapies may have different effects on the response of primary and distant tumor sites. For example, vaccination is an emerging area of research that has shown promising results for the future of immune therapy. Activated whole tumor cell vaccines can enhance the immune response induced by radiation therapy by inhibiting immune checkpoint molecules such as PD-1 (Rückert et al. 2021b). Recent studies have emphasized the importance of increased levels of novel oxidative phosphorylation (OXPHOS) in tumors, which is related to the efficacy of immune therapy (Pustylnikov et al. 2018; Chamoto et al. 2017), Anti-PD-1 antibodies or radiation therapy can increase OXPHOS levels (Chamoto et al. 2017; Ashton et al. 2018; Le et al. 2018). Therefore, combined treatment with radiation therapy and OXPHOS inhibitors may be an effective strategy against PD-1 resistance in esophageal cancer (Chen et al. 2020).

Understanding the immune mechanisms of radiation therapy is crucial in unleashing the potential and revolutionizing cancer treatment based on immune-combined radiation therapy. Through studying the immune mechanisms of radiation therapy, it contributes to the design and implementation of immune-combined radiation therapy, providing new avenues for personalized and targeted treatment of tumors.

## Low-dose radiotherapy enhances the efficacy of immune-combined radiation therapy: updates and challenges

The adverse tumor microenvironment, such as vascular barriers, lack of appropriate cytokines and immune suppression factors, hinders T cell infiltration and thus impairs the effectiveness of immune therapy (Motz and Coukos 2013; Sharabi et al. 2015). High-dose radiation therapy, whether delivered conventionally or through stereotactic body radiation therapy (SBRT), can induce the production and release of cytokines and chemokines, creating an inflammatory microenvironment that promotes T cell infiltration and immunogenic cell death (McLaughlin et al. 2020; Darragh et al. 2018; Donlon et al. 2021). However, high-dose radiation therapy alone may not fully address immune suppression factors, such as inhibitory tumor microenvironment. Recent studies suggest that low-dose radiotherapy (LDRT) holds promise in addressing these limitations and improving the outcome of immune therapy. LDRT is typically defined as fractionated doses of 0.5–2 Gy and total doses of 1–10 Gy (Patel et al. 2021a; Menon et al. 2019; Yin et al. 2020; Barsoumian et al. 2020), which are generally considered non-cytotoxic but have the potential to modulate the tumor microenvironment and enhance T cell infiltration.

LDRT demonstrates additional synergistic effects supported by radiobiological data, including the phenomenon of radiation hypersensitivity, where even low doses of X-rays as low as 0.1 Gy can effectively induce tumor cell death. Dynamic microscopy imaging studies have confirmed the effectiveness of LDRT in real-time visualization experiments, providing evidence for its potential as a treatment strategy (Joiner et al. 2001; Short et al. 1999; Marples et al. 1997). In clinical research, the combination of LDRT with chemotherapy has shown significant tumor control rates, particularly when using doses that can induce radiation hypersensitivity (Mantini et al. 2012; Regine et al. 2007; Balducci et al. 2012). By combining LDRT with other treatments such as chemotherapy or immunotherapy, the synergistic effects can be utilized to improve cancer treatment outcomes.

LDRT has the potential to reshape the tumor microenvironment and facilitate T cell homing, providing new possibilities for enhancing immunotherapy. While high-dose fractionated radiotherapy has demonstrated in-situ vaccine effects and ex vivo effects, direct evidence for similar effects from low-dose radiation therapy is still limited. LDRT shows promise in patients lacking tumor-infiltrating CD8 T cells, as it can modulate the tumor microenvironment and facilitate T cell recruitment (Klug et al. 2013; Liu et al. 2019). LDRT plays a particularly vital role in the preparatory stage of combining immunotherapy to induce T cell homing.

Animal experiments have shown that low-dose radiotherapy (LDRT) combined with immune checkpoint inhibitors (ICIs) can better control tumors, including ovarian cancer, breast cancer, and gliomas, among others (Herrera et al. 2022a, b; Patel et al. 2021b). Among them, the combination of low-dose radiation therapy and PD-1 blockade achieved complete tumor remission in 85.7% of mice (Yin et al. 2020). Low-dose radiation (0.5–2 Gy) demonstrated evidence of tumor microenvironment (TME) reprogramming and induction of M1 macrophage polarization in a mouse model of localized pancreatic neuroendocrine tumors (Deloch et al. 2019; Nadella et al. 2018). iNOS-positive M1 macrophages play a crucial role in recruiting effector T cells, promoting tumor vascular normalization and inflammation, and facilitating T cell infiltration into the tumor microenvironment (Klug et al. 2013; Prakash et al. 2016; Palma et al. 2013). Combining high-dose irradiation of the primary tumor, low-dose radiation therapy of metastatic lesions, and immune checkpoint inhibitors in a mouse lung cancer model can effectively control metastatic tumors through immune response and downregulation of immunosuppressive factor TGF-β (Barsoumian et al. 2020).

Clinical studies have shown that LDRT, as a new adjuvant therapy for pancreatic cancer patients, can increase the ratio of iNOS macrophages to CD8 T cells and reduce tumor vessel diameter (Klug et al. 2013). Clinical trials of LDRT combined with immunotherapy in patients with peritoneal metastatic ovarian cancer have demonstrated that LDRT induces transient tumor inflammation, making it sensitive to immunotherapy (Herrera et al. 2022a). Conversely, LDRT may have inhibitory effects on inflammatory lesions driven by benign inflammation or autoimmune T cell-driven diseases (Rödel et al. 2012a, b). Further clinical research is needed to compare the impact of LDRT on the tumor microenvironment and determine the optimal dosage range for macrophage remodeling and enhanced T cell infiltration.

In the exploration of improving the efficacy of immunotherapy, the combination of low-dose radiotherapy (LDRT) and high-dose radiation therapy has emerged as a promising treatment strategy. Although LDRT cannot directly kill tumor cells, it has the potential to activate immune cells and modulate the tumor microenvironment, thereby enhancing the effectiveness of immunotherapy. Recent clinical trials have investigated the combination of stereotactic body radiation therapy (SBRT) and the immune checkpoint inhibitor ipilimumab in the treatment of advanced metastatic lesions. The results suggest that tumors exposed to LDRT exhibit higher treatment responses compared to distant tumors (Welsh et al. 2019). The combination of high-dose radiation and LDRT represents a new treatment approach, where high-dose radiation promotes antigen release and presentation, and activates immune cells, while LDRT facilitates immune cell infiltration into distant tumor microenvironments (Menon et al. 2019). The results of a non-randomized phase II trial combining LDRT, high-dose radiation, and immunotherapy showed that disease regions exposed to LDRT were more likely to generate local responses (Patel et al. 2021). Future clinical trials should compare the efficacy of different LDRT regimens in combination with immune checkpoint inhibitors (ICIs) to determine the optimal approach. These strategies can serve as palliative treatment options for refractory patients, reshaping the tumor microenvironment and inducing new anti-tumor responses. The combination of high-dose SBRT triggering in-situ vaccine effects at limited metastatic sites, along with LDRT targeting other metastatic sites to activate T cells, holds the potential to maximize distant effects.

Low-dose radiotherapy (LDRT) has emerged as a promising approach to overcome the limitations of high-dose radiation therapy. High radiation doses at the primary site of esophageal cancer can lead to serious adverse effects such as bleeding and perforation. Therefore, combining high-dose radiation therapy for distant metastases with low-dose radiation therapy at the primary site may be a new treatment method for advanced esophageal cancer. Although further research is needed to fully explore its capabilities, LDRT has the potential to become an important avenue for future investigations. By optimizing the use of radiation therapy, particularly at low doses, we can improve treatment effectiveness and explore new possibilities in cancer management.

## Conclusion

The potential of radioimmunotherapy in improving the prognosis of esophageal cancer patients is exciting. Large-scale studies are currently underway to explore various aspects, such as radiation dosage, fractionation, irradiation sites and techniques, timing and duration of combined therapy, and the selection of immunotherapy agents, with the aim of uncovering its anti-tumor mechanisms and predictive biomarkers to enhance treatment efficacy. Radioimmunotherapy may present a new approach for patients with locally advanced or metastatic esophageal cancer, especially when combined with ICIs and low-dose radiation, as it can induce a systemic anti-tumor response. As immunotherapy plays an increasingly important role in solid tumor treatment, leveraging the synergistic advantages between radiation therapy and immunotherapy could open up new avenues for the design of combination immunotherapy.

## Data Availability

Not applicable.

## Abbreviations

APCs: Antigen-presenting cells
ctDNA: Circulating tumor DNA
DAMPs: Damage-associated molecular patterns
DCs: Dendritic cells
ESCC: Esophageal squamous cell carcinoma
ICIs: Immune checkpoint inhibitors
LDRT: Low-dose radiotherapy
MDSCs: Myeloid-derived suppressor cells
MDSCs: Myeloid-derived suppressor cells
MHC: Major histocompatibility complex
OS: Overall survival
pCR: Pathologic complete response
PD‐1: Programmed cell death protein 1
PD‐L1: Programmed cell death protein ligand 1
PFS: Progression-free survival
RT: Radiation therapy
SBRT: Stereotactic body radiation therapy
SRS: Stereotactic radiosurgery
TAAs: Tumor-associated antigens
TAMs: Tumor-associated macrophages
TGF-β: Transforming growth factor-beta
TILs: Tumor-infiltrating lymphocytes
TMB: Tumor mutational burden
TME: Tumor microenvironment
TRAEs: Treatment-related adverse events
Tregs: Regulatory T cells
